# An Automated Perifusion System for Modifying Cell Culture Conditions over Time

**DOI:** 10.1186/s12575-016-0049-7

**Published:** 2016-11-21

**Authors:** Nicholas B. Whitticar, Elisha W. Strahler, Parthiban Rajan, Savas Kaya, Craig S. Nunemaker

**Affiliations:** 1 Diabetes Institute, Ohio University, Athens, OH USA; 2 Department of Biomedical Sciences, Heritage College of Osteopathic Medicine, Ohio University, Athens, OH USA; 3 School of Electrical Engineering & Computer Science, Ohio University, Athens, OH USA

**Keywords:** Diabetes, Islets, Beta cells, Perifusion, Automated, Calcium, Florescence imaging, Insulin, Absorbance, Syringe pump, Oscillations, Glucose

## Abstract

**Background:**

Cells are continuously exposed to changes in their environment. Endocrine systems, in particular, communicate by rhythms and feedback loops. In this study, we developed an automated system to produce such conditions for cultured cells in a precisely timed manner. We utilized a programmable pair of syringe pumps for inflow and a peristaltic pump for outflow to create rhythmic pulses at 5-min intervals in solutions that mimic the endogenous patterns of insulin produced by pancreatic islets as a test case.

**Results:**

This perifusion system was first tested by measuring trypan blue absorbance, which was intermittently added and washed out at 3:3 and 2:3 min (in:out). Absorbance corresponded with patterns of trypan blue delivery. We then created patterns of forced oscillations in islets by intermittently switching between solutions containing 28 millimolar (mM) glucose (producing high levels of intracellular calcium ([Ca^2+^]_i_) and insulin secretion) and 28 mM glucose + calcium-channel blocker nifedipine (producing low levels of [Ca^2+^]_i_ and insulin secretion). Forced perifusion effects were monitored by fura-2 AM fluorescence measurements of [Ca^2+^]_i_. Islets showed uniform oscillations in [Ca^2+^]_i_ at time intervals consistent with the perifusion pattern, mimicking endogenous pulsatility.

**Conclusions:**

This study highlights a valuable method to modify the environment of the cell culture over a period of hours to days.

## Background

Traditional and automated perifusion systems have long been used to flow chemical solutions over living tissue [1–4]. The fluids used can be perifused at physiologically relevant flow rates and temperatures to mimic in vivo conditions and to remove unwanted environmental variables such as osmotic and mechanical stressors [2, 4, 5]. These systems are especially useful for perifusing hormones over immobilized cells, which allows the experimenter to collect the cellular outflow for analysis. Examples that rely on these systems include the release of anterior pituitary hormones when stimulated by other hormones or chemicals [1–3]. Perifusion is also widely used in diabetes research when studying pancreatic islets in vitro [4, 6]. The ability to perifuse chemicals over cells for long periods of time in an automated fashion can be useful for a variety of these studies. Automation of the traditional perifusion system allows experiments to be carried out using rapid and systematic alternation of chemicals on a timed basis to further mimic in vivo hormonal signaling. Certain biological functions such as endogenous oscillations in islets and anterior pituitary hormone exposure are perfectly suited for such a system. It also allows cells to be exposed to multiple conditions over a period of hours to days without intervention by the researcher.

Previous studies in our lab have used a peristaltic pump to flow differing glucose solutions over pancreatic islets while monitoring intracellular calcium ([Ca^2+^]_i_) in response to glucose [7]. When studying the long term effect of differing cell culture compositions on the islets over the course of several days, the automated system becomes necessary. Using the SyringePumpProV1 computer system, each individual pump can be set to flow at a given rate and time, which eliminates the need for the experiment to be monitored.

To evaluate the efficacy of an automated perifusion system, a trypan blue absorbance analysis and calcium florescence imaging were utilized. The first study used the automated pumps to switch between a trypan blue solution and water with the output solution collecting in a 96-well plate. The absorbance results showed that two solutions can be used to provide an oscillatory solution bath over the cells. A second study demonstrated the effect of switching between a 28 mM glucose solution to stimulate calcium influx and a 28 mM glucose solution containing 1.25 μM nifedipine to prevent calcium influx on pancreatic islets. The changes in [Ca^2+^]_i_ were measured using fura-2 AM calcium florescence imaging. These results showed that changes in [Ca^2+^]_i_ corresponded with the times the solutions were alternated. Both experiments indicate that the system can be used without human supervision to provide automated alterations in the environment of cultured cells.

## Methods

### Automated Perifusion System Design

**Fig. 1:**
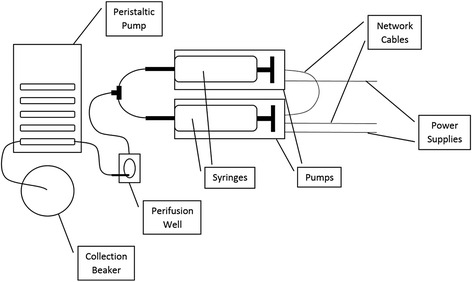
Schematic of the automated syringe system used for experiments. Note that the collection beaker was replaced with a 96 well plate for the trypan blue experiments (Figs. 2 and 3)

The automated syringe pump perifusion system (New Era Pump Systems, Inc., Farmingdale, NY, Model NO. NE-500) is a timed system that pumps solutions out in exact amounts in a perfectly timed manner that corresponds to a code created by the computer program SyringePumpProV1 (SyringePumpPro, Gawler, South Australia, Version 1.6.4.7). After the syringes are loaded into the pump apparatus, the syringes are each connected to tubing. The tubing exiting each pump comes together at a Y-junction with a single tube entering into an open diamond bath imaging chamber (Warner Instruments, Hamden CT, Cat: 64–0288). After exiting the cell chamber, tubing connects to the chamber and is threaded into a minipuls 2 peristaltic pump (Gilson, Middleton, WI) which removes the fluid from the well. A detailed protocol of the pump set-up and pump program can be found in Appendix below. A schematic of the automated syringe pump system can be found below in Fig. 1.

### Trypan Blue Tests

To test that the automated perifusion system performs as desired, initial tests using trypan blue dye (Life Technologies, Eugene, OR) and deionized water were carried out to show that the automated syringe pump perifusion system can pump the liquids out at a controlled rate. To begin, a 0.4% trypan blue dye was diluted with deionized water using a 1:5 ratio (2 mL dye to 8 mL H_2_O). The solution was then transferred into a 10 cc BD syringe and placed into the first pump of the automated syringe system. An identical syringe was then filled with 10 mL of deionized water and placed into the second pump. The tubing entered a microfluidic cell chamber with a peristaltic pump system set at 200 μL/min to remove fluid from the well. After the peristaltic pump system the end of the tubing was placed into a well in a Costar 96 well plate (Corning Inc., Corning, NY, Product #3596). The tubing was moved to the next well every minute for 36 min while skipping every other row. Using The SyringePumpProV1 computer program, the pump with the water was set to run at 200 μL/min for three minutes while the pump with the dye remained paused. Then the pump with the water shut off and the pump with the dye cycled on for three minutes during Experiment 1 and two minutes during Experiment 2. These cycles continued for 36 min until three rows on the 96-well plate were filled. 100 μL out of each well was then pipetted into the well one row below the existing well (the well that was skipped when filling) to make a set of duplicate wells. The absorbance of each well was read using the FLUOstar Optima microplate reader (BMG Labtech Inc., Cary, NC). On the layout tab, each well sample was matched with the corresponding well in the row under it to create an average. The excitation filter was then set on an absorbance of 595 nm to measure the samples [8].

### Forced Islet Oscillations

To show that the automated syringe perifusion system works in actual implicative experimentation, a forced oscillation test of pancreatic islets was carried out. Pancreatic islets were isolated from adult outbred male CD-1 mice and cultured as previously described by Carter et al. [9]. These islets were then incubated in a solution with 1 μL of fura-2 AM dye (Life Technologies, Eugene, OR) and 1 mL of a modified Krebs Ringer Buffer solution containing 11 mM glucose for 30 min [9]. Forced oscillations of dye-bound [Ca^2+^]_i_ derive primarily from the insulin-producing beta cells, which produce the majority output response to glucose in the pancreatic islets [10, 11]. In this experiment, a solution of the calcium channel blocker nifedipine (Sigma-Aldrich, St. Louis, MO) (5 μL of 10 mM nifedipine in 40 mL of 28 mM glucose) and a high-glucose solution (28 mM) were applied in an alternating fashion over pancreatic islets in a perifusion chamber. The nifedipine acts as a calcium channel blocker to inhibit the function of beta cells in the islets (and decrease overall calcium influx and corresponding insulin release), while the 28 mM glucose activates beta cells in the islets (increasing calcium channel activity and insulin release) [11]. Alternating infusion of these two solutions is designed to cause a wave-like activation and de-activation of islet calcium channel activity.

To start, each solution was filled into a 60 cc BD syringe. Using the SyringePumpProV1 computer program, the pump with the nifedipine was set to run at 200 μL/min for two minutes while the pump with the glucose remained paused. Then the pump with the nifedipine shut off and the pump with the 28 mM glucose cycled on for three minutes. These cycles continued on loop for the entirety of the 1 h and 10 min experiment. The islets were placed into the chamber at the 15 min mark of the experiment, so that each solution had cycled through a few times. The tubing exiting each syringe passed through an in-line heater (Warner Instruments, Hamden, CT, Cat: 64–0103) to bring the solutions to a physiologically relevant temperature (~32–37° Celsius). After exiting the well, fluid was removed by a separate peristaltic pump system set at 200 μL/min.

### [Ca^2+^]_i_ Imaging

Fura-2 AM fluorescence imaging was utilized to measure [Ca^2+^]_i_ levels. The perifused solution first passed through an in-line heater into an open diamond bath imaging chamber (Warner Instruments, Cat: 64–0288) which was mounted using a stage adapter (Warner Instruments, Cat: 64–0298). Observation of islets was performed using a Hamamatsu ORCA-Flash4.0 digital camera (Hamamatsu Photonics K.K., Hamamatsu City, Japan, Model C11440-22CU) mounted on a BX51WIF fluorescence microscope with a 10× objective (Olympus, Tokyo, Japan). Excitation light was provided by a xenon burner supplied to the image field through a light pipe and filter wheel (Sutter Instrument Co., Novato CA, Model LB-LS/30) with a Lambda 10–3 Optical Controller (Sutter Instrument Co., Novato, CA, Model LB10-3-1572). Images were taken sequentially from 340 nm to 380 nm excitation to produce each [Ca^2+^]_i_ ratio from emitted light at 510 nm. Data were analyzed using cellSens Dimension 1.13 imaging software (Olympus, Tokyo, Japan).

## Results

### Trypan Blue Tests

**Fig. 2:**
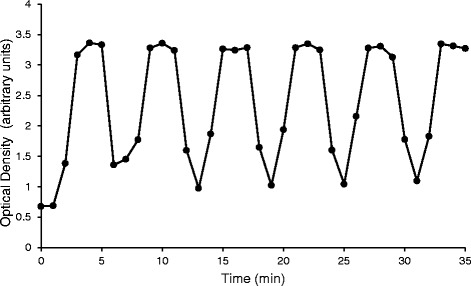
Absorbance readings from the trypan blue (3 min on:3 min off) test. The syringe pump alternated between pumping 3 min of trypan blue then 3 min of water for the entirety of the experiment. The y-axis is labeled as the arbitrary unit optical density, which is a measurement of absorption at 595 nm wavelength

**Fig. 3:**
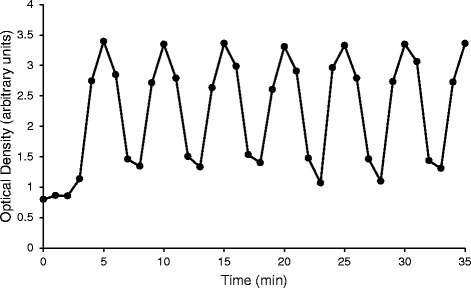
Absorbance readings from the trypan blue (2 min on:3 min off) test. The syringe pump alternated between pumping 2 min of trypan blue then 3 min of water for the entirety of the experiment. The y-axis is labeled as the arbitrary unit optical density, which is a measurement of absorption at 595 nm wavelength

Data from the trypan blue absorbance analysis was graphed as a function of optical density vs time which demonstrates the oscillatory pattern desired from the syringe pump system. The difference between running a 3:3 and 2:3 can be shown in Figs. 2 and 3, respectively. Note that the 3:3 pattern produces a longer plateau phase (Fig. 2, three points near peak indicating a longer period of heightened dye concentration) compared to the 2:3 pattern (Fig. 3, a single peak point). The differences between 2:3 and 3:3 patterns demonstrate that the pump system can produce subtle changes in the ‘plateau fraction’ of oscillations, which are indicative of very sensitive biological responses to small changes in nutrient load in pancreatic islets [12, 13]. Therefore, the syringe pump system can be used to create an experimentally useful oscillatory pattern of solutions using multiple pumps.

### Forced Islet Oscillations

**Fig. 4:**
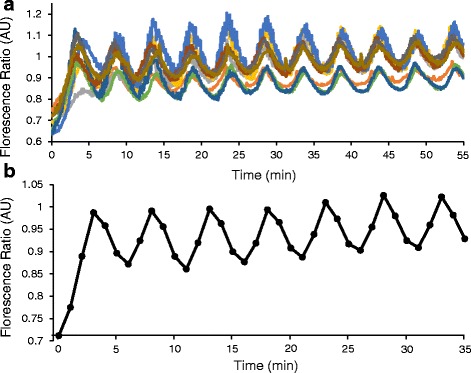
Forced oscillations in islets. **a** Example of glucose-stimulated [Ca^2+^]_i_ results from a forced oscillations experiment containing *N* = 9 islets over a period of 55 min. The y-axis is labeled as the ratio of excitation and emission fluorescence (340/380 nm) to indicate changes in islet [Ca^2+^]_i_ throughout the experiment. **b** Time averaged results of the forced oscillation experiment. The average florescence ratio of the islets was calculated and time averaged in one minute intervals to mirror the trypan blue timed studies

We next examined whether the automated perifusion system could manipulate physiological responses of biological tissue in a similar manner. We examined changes in [Ca^2+^]_i_ in pancreatic islets isolated from common outbred CD-1 mice as a test case. The results of forced perifusion on several islets are shown in Fig. 4a using measurements of [Ca^2+^]_i_ at 5-s intervals. Average florescence ratios of all islets were calculated and time averaged in one minute intervals as shown in Fig. 4b. This figure reflects the trypan blue 2:3 ratio study, which demonstrates that culture conditions containing biological tissues can be altered just as quickly and accurately as for inert dyes. Thus, we provide proof-of-priniciple that the syringe pump system can be used to create programmed changes in cell culture conditions over long periods of time.

## Discussion

The forced oscillation experiments performed on pancreatic islets demonstrate that the automated syringe pump perifusion system can effectively infuse solutions in a timed manner and rate. The automated perifusion system produces the same results that a manual perifusion system would but allows automated and programmable alteration of media conditions over hours to days to weeks as needed. Many applications can stem from this new automated syringe pump system. As shown, this system can function to carry out pancreatic islet experimentation flowing various solutions over cells. However, as described, this syringe pump also has the potential to benefit other experiments that involve perifusion of various solutions over cells for short or long periods of times.

Other automated perifusion systems do already exist, however, it is the variation of systems and set-up that can further experimentation in many different areas. For instance, Hsiao et al. created an automated perifusion system containing an automated fluidic control unit with a microfluidic chip to view calcium signal transduction relating to taste sensing enteroendocrine cells [14]. Similarly to the automated syringe pumps, this system allows accurate and reliable flow of solutions over live cells [14]. As a limitation, the system must be monitored to change flow rates using different pressures of compressed air whereas our system can quickly change flow rates and solutions following a computer program [14]. Another example of an automated perifusion system was utilized by Anderson et al. for the purpose of examining synaptic function [15]. The system was designed to allow entire synaptic plasticity experiments to be run in a fully automated fashion [15]. Automatic changing of many solutions with accurate timing permitted integration of automated electrical stimulation and data acquisition [15]. An additional advantage of our system is that the New Era Pump Systems’ syringe pumps are designed to be linked in series, permitting multiple syringe pumps to be programmed to operate in any order for any duration. Variety in pumping apparatuses and cell chambers allow similar systems to be applied to a variety of cell culture experiments.

Perhaps the most useful application for the automated syringe pump system is in the field of endocrinology. Many hormones travel through blood vessels and effect cells in a similar fashion that perifusion systems provide. The traditional use of a peristaltic pump does not allow pulsatile release or automated switching of hormones pumping through the system. However, this system can be programed to apply solutions in pulses, which can be useful for hormones released in this fashion such as insulin, luteinizing, and growth hormones [2, 3, 16]. The long-term goal of our work is to manipulate different cellular processes in the glucose-stimulated insulin secretion pathway to determine which of the multiple oscillatory systems in beta-cells may be important to long-term viability and function [17]. Long term studies can also be conducted to show the typical slow effect of hormonal changes such as in female ovulatory cycles [18]. In studies relating to pituitary hormones, many different chemicals can be added automatically for long periods to utilize these long lasting tissues to their full extent. Perifused pituitary tissues are known to release much more hormone when compared to static cultures [3]. In an example relating to pancreatic islets, the cells can be studied overnight without supervision to see the long term effect of perifusing different solutions. The calcium influx response or total insulin release of the β cells can then be analyzed. The pulsatile patterns we created in islets are similar to endogenous insulin pulses released into the portal system that directly target the liver. Thus, the same simulated pulsatility could be valuable to the study of hepatocytes [19, 20]. The automation of the typical peristaltic perifusion system opens up many new possibilities for live culture studies.

The current perfusion system is being integrated to a custom 3D-engineered microfluidics platform that streamlines the cell introduction, fluid delivery and collection along with in-situ sensors to monitor culturing conditions and specific ionic or molecular signatures (Zn and Glycolysis precursors). This is expected to result in a more compact and accurate perfusion system with direct electrical readout of analytes in the culturing solution as well as outflow. In addition, it can also provide a platform for more complex cell treatment/culturing scenarios (up to a dozen different solutions with fast switching times), different cell types, longer episodic experiments (over hours to days to weeks if necessary) and shorter data analysis cycles. Preliminary work with such 3D printed microfluidic platforms are currently underway and will be published in a subsequent article.

## Conclusions

Our findings show that the automated syringe pump perifusion system can deliver complex patterns of media/nutrients over time to cell cultures. This system opens up the possibility of simulating in vivo physiological conditions for ex vivo cells over extended periods of time, such as alternating between meals and fasting periods for pancreatic islets, simulating pulsatile insulin delivery to cultured liver tissue, or even mimicking the complex milieu of hormones of the menstrual cycle for gonadal tissue. Thus, many applications can stem from this new automated syringe pump system’s ability to simulate in vivo physiological conditions in ex vivo settings.

## Appendix

### Automated Perfusion Protocol

1. Plug USB cable into the computer and attach the network cable adapter labeled “CBL-PC-PUMP”. The network cable goes into first pump where it is labeled “computer” on the bottom side. This is now “Pump 00”. Connect the next network cable from slot labeled “pump network” on pump 00 to the next pump in the slot labeled “computer”. This is now “Pump 01”. Plug in power supplies to both pumps (Pumps should beep upon power being connected).
2. Label and fill syringes to appropriate level with no bubbles. (Tap on lab bench to loosen bubbles then expel air from needle).
3. Place syringes in apparatus placing head of plunger in vice and tighten (screw direction is opposite of standard).
4. Pull up and spin tab over barrel of syringe to secure in place.
5. Connect hosing to the end of each syringe coming together at a “Y” junction. A single hose should then go completely through the heating apparatus and into the tube connected to the well. The peristaltic pump outflow should also be assembled and set to match the flow rate that will be programed.
6. Squeeze white tab in on the side of the syringe slide and move until the second fluid needed is seen at the “Y” junction. Repeat this with the remaining pump containing the fluid that will be used when loading the islets. This time the slide should be moved until the fluid is seen filling up the well enough to make a meniscus with the objective lens.
7. Open “SyringePumpProV1” on computer. Make sure pump 00 and pump 01 are loaded in pump worksheet. If not, reassure network and power cables are fully inserted and restart the program.
8. Click “PPL” tab on top of screen, PPL Creator, Standard.
9. See *How to Create Program* below.
10. After making protocol for pump 00, right click on pump 00, upload PPL file. Allow program to run through the protocol until you hear a beep. Click “OK” on the popup which sometimes comes up behind the current window. Then, right click on pump 00 and click “refresh values”.
11. Repeat steps 8–10 for pump 01.
12. Click ‘run all’ on right of screen and turn on the peristaltic pump for outflow. The system must be balanced so the outflow nail is high enough for the well not to dry out over long periods.
13. To pause the program, click “Stop/Pause” once. To resume, click “Start” again. To restart the program, click “Stop/Pause” twice and make sure the “Amount Dispensed” column resets to 0.

### How to Create Program

1. Step 1 should always be “loop start”.
2. Each phase number can be programed with a unique function.
  - Rate for time allows you to enter the rate of fluid you need pumped for a set time. Pumping rate units can be changed and pumping direction must be set on infuse.
  - Pause stops the pump for a set amount of time. The maximum time allowed is 99 s, if the time needed is greater, you must add another pause phase.
  - Jump to phase allows you to enter a phase number to make a continuous loop to.
  *When switching between which pump is working, there must be a 1 s delay. For example, if pump 00 is running for 60 s, pump 01 must pause for 61 s before starting to pump.
3. After the desired program is made, scroll to the right and assure the correct syringe manufacturer and size are set. Since this is the program for Pump 00, check to make sure it says “0” next to “pump address”.
4. Change the spreadsheet you are on to the tab labeled “PPL”. Then click file, save as and choose the folder location. Change the file type to “Text (Tab Delimited)” and add the suffix “. PPL” to the file name you chose. Click Save, Yes, Ok.

## Abbreviations

[Ca^2+^]_i_: Intracellular calcium
mM: millimolar

## References

[1] Temamogullari NE, Nijhout HF, Reed M C (2016). Mathematical modeling of perifusion cell culture experiments on GnRH signaling. Math Biosci.

[2] Becker K, Conway S (1992). A novel hypothalamic-dispersed pituitary co-perifusion model for the study of growth hormone secretion. Brain Res.

[3] Hassan HA, Merkel RA (1994). Perifusion model system to culture bovine hypothalamic slices in series with dispersed anterior pituitary cells. In Vitro Cell Dev Biol Anim.

[4] Morris C, Banks DJ, Gaweda L, Scott S, Zhu XX, Panico M, Georgiou P, Toumazou C (2011). A robust microfluidic in vitro cell perifusion system. Conf Proc IEEE Eng Med Biol Soc.

[5] Walker GM, Zeringue HC, Beebe DJ (2004). Microenvironment design considerations for cellular scale studies. Lab Chip.

[6] Heileman K, Daoud J, Hasilo C, Gasparrini M, Paraskevas S, Tabrizian M (2015). Microfluidic platform for assessing pancreatic islet functionality through dielectric spectroscopy. Biomicrofluidics.

[7] Nunemaker CS, Dishinger JF, Dula SB, Wu R, Merrins MJ, Reid KR, Sherman A, Kennedy RT, Satin LS (2009). Glucose metabolism, islet architecture, and genetic homogeneity in imprinting of [Ca2+](i) and insulin rhythms in mouse islets. PLoS One.

[8] Uliasz TF, Hewett SJ (2000). A microtiter trypan blue absorbance assay for the quantitative determination of excitotoxic neuronal injury in cell culture. J Neurosci Methods.

[9] Carter JD, Dula SB, Corbin KL, Wu R, Nunemaker CS (2009). A Practical Guide to Rodent Islet Isolation and Assessment. Biol Proced Online.

[10] Cabrera O, Berman DM, Kenyon NS, Ricordi C, Berggren P-O, Caicedo A (2006). The unique cytoarchitecture of human pancreatic islets has implications for islet cell function. Proc Natl Acad Sci U S A.

[11] Ramadan JW, Steiner SR, O’Neill CM, Nunemaker CS (2011). The central role of calcium in the effects of cytokines on beta-cell function: implications for type 1 and type 2 diabetes. Cell Calcium.

[12] Nunemaker CS, Bertram R, Sherman A, Tsaneva-Atanasova K, Daniel CR, Satin LS (2006). Glucose modulates [Ca2+]i oscillations in pancreatic islets via ionic and glycolytic mechanisms. Biophys J.

[13] Corbin KL, Waters CD, Shaffer BK, Verrilli GM, Nunemaker CS (2016). Islet hypersensitivity to glucose is associated with disrupted oscillations and increased impact of proinflammatory cytokines in islets from diabetes-prone male mice. Endocrinology.

[14] Hsiao Y-H, Hsu C-H, Chen C. A High-Throughput Automated Microfluidic Platform for Calcium Imaging of Taste Sensing. Molecules. 2016;21:896. 10.3390/molecules21070896 PMC6273845 27399663

[15] Anderson WW, Fitzjohn SM, Collingridge GL (2012). Automated multi-slice extracellular and patch-clamp experiments using the WinLTP data acquisition system with automated perfusion control. J Neurosci Methods.

[16] Armstrong SP, Caunt CJ, Fowkes RC, Tsaneva-Atanasova K, McArdle CA (2010). Pulsatile and Sustained Gonadotropin-releasing Hormone (GnRH) Receptor Signaling. J Biol Chem.

[17] Heart E, Smith PJS (2007). Rhythm of the beta-cell oscillator is not governed by a single regulator: multiple systems contribute to oscillatory behavior. Am J Physiol Endocrinol Metab.

[18] Christian CA, Moenter SM (2010). The Neurobiology of Preovulatory and Estradiol-Induced Gonadotropin-Releasing Hormone Surges. Endocr Rev.

[19] Matveyenko AV, Veldhuis JD, Butler PC (2008). Measurement of pulsatile insulin secretion in the rat: direct sampling from the hepatic portal vein. Am J Physiol Endocrinol Metab.

[20] Najjar SM, Yang Y, Fernström MA, Lee S-J, Deangelis AM, Rjaily GAA, Al-Share QY, Dai T, Miller TA, Ratnam S, Ruch RJ, Smith S, Lin S-H, Beauchemin N, Oyarce AM (2005). Insulin acutely decreases hepatic fatty acid synthase activity. Cell Metab.

